# Cost-Effectiveness Analysis of Community Active Case Finding and Household Contact Investigation for Tuberculosis Case Detection in Urban Africa

**DOI:** 10.1371/journal.pone.0117009

**Published:** 2015-02-06

**Authors:** Juliet N. Sekandi, Kevin Dobbin, James Oloya, Alphonse Okwera, Christopher C. Whalen, Phaedra S. Corso

**Affiliations:** 1 Department of Epidemiology and Biostatistics, College of Public Health, University of Georgia, Athens, Georgia, United States of America; 2 Department of Health Policy and Management, College of Public Health, University of Georgia, Athens, Georgia, United States of America; 3 Department of Epidemiology and Biostatistics, School of Public Health, College of Health Sciences, Makerere University, Kampala, Uganda; 4 School of Medicine, College of Health Sciences, Makerere University, Kampala, Uganda

## Abstract

**Introduction:**

Case detection by passive case finding (PCF) strategy alone is inadequate for detecting all tuberculosis (TB) cases in high burden settings especially Sub-Saharan Africa. Alternative case detection strategies such as community Active Case Finding (ACF) and Household Contact Investigations (HCI) are effective but empirical evidence of their cost-effectiveness is sparse. The objective of this study was to determine whether adding ACF or HCI compared with standard PCF alone represent cost-effective alternative TB case detection strategies in urban Africa.

**Methods:**

A static decision modeling framework was used to examine the costs and effectiveness of three TB case detection strategies: PCF alone, PCF+ACF, and PCF+HCI. Probability and cost estimates were obtained from National TB program data, primary studies conducted in Uganda, published literature and expert opinions. The analysis was performed from the societal and provider perspectives over a 1.5 year time-frame. The main effectiveness measure was the number of true TB cases detected and the outcome was incremental cost-effectiveness ratios (ICERs) expressed as cost in 2013 US$ per additional true TB case detected.

**Results:**

Compared to PCF alone, the PCF+HCI strategy was cost-effective at US$443.62 per additional TB case detected. However, PCF+ACF was not cost-effective at US$1492.95 per additional TB case detected. Sensitivity analyses showed that PCF+ACF would be cost-effective if the prevalence of chronic cough in the population screened by ACF increased 10-fold from 4% to 40% and if the program costs for ACF were reduced by 50%.

**Conclusions:**

Under our baseline assumptions, the addition of HCI to an existing PCF program presented a more cost-effective strategy than the addition of ACF in the context of an African city. Therefore, implementation of household contact investigations as a part of the recommended TB control strategy should be prioritized.

## Introduction

Tuberculosis (TB) disease continues to pose a serious public health threat despite decades of sustained control efforts worldwide. The World Health Organization (WHO) estimates that nearly 9 million new cases of TB occur while 2 million people die annually [1]. Of the new cases, nearly 80% reside in the 22 high-burden countries including Uganda. In 2011, Uganda’s estimated annual TB incidence rate was 330/100,000 with a death rate of 5.3% [2]. Deaths from TB disease are associated with a high economic burden as projected by a World Bank study on the economic benefit of TB control, that the cost of TB—related deaths (including HIV co-infection) in Sub-Saharan Africa from 2006 to 2015 would be US$ 519 billion when there is no effective TB treatment and control as prescribed by WHO’s Stop TB Strategy [3].

Case detection is a cornerstone of the TB control strategy recommended by the WHO; yet the standard passive case finding (PCF) approach has not achieved universal success in detecting all cases. Globally, it is estimated that nearly 30% of the new TB cases remain undetected [4]. Moreover the cases detected through PCF experience long delays prior to diagnosis thus continue to transmit disease while they are still in the community. Alternative strategies of case detection such as community active case finding (ACF) and household contact investigations (HCI) have been shown to be effective [5,6]. However, very few studies have evaluated the cost-effectiveness of these strategies compared to the standard PCF [7,8].

Over the past two decades, economic evaluation studies of broad tuberculosis (TB) control interventions have become increasingly common. These studies have focused on areas such as screening for latent TB infection [9–11], screening for active TB among contacts [7,12], length and type of drug regimens [13], diagnostic strategies [14–18], treatment of multidrug resistant TB [19–21] and delivery of TB care [22–26]. But, far fewer studies have been published on case detection strategies of active TB even though it is a core component of the current TB control strategy [7,8,27–29]. The Uganda Health Sector Strategic Plan recommends that the Uganda Ministry of Health must make evidence-based decisions to allocate scarce resources among communicable disease health programs [30] however, a dearth of evidence exist on the costs and health effects of TB case finding strategies. Therefore, the objective of this study was to determine whether the addition of Active Case Finding or Household Contact Investigation to Passive Case Finding compared with standard Passive Case Finding alone represent cost-effective strategies for detection of true TB cases in urban Uganda.

## Methods

### Ethical Considerations

The primary study was approved by the University of Georgia Institutional Review Board, Makerere University School of Public Health Higher Degrees, Research and Ethics Committee, and Uganda National Council for Science and Technology. Official permission was obtained to utilize National TB program clinic records. Written informed consent was obtained from all participants.

This study utilized a static decision analytic model to evaluate the cost and cost-effectiveness of three strategies for detecting TB cases including the current WHO recommended standard PCF alone and in combination with either ACF or HCI. Using incremental cost-effectiveness analysis, we compared the additional cost in 2013 US dollars per true TB case detected across the three strategies.

### Study Setting and Target Population

The study was based in Kampala, which represents an urban African city with a high prevalence of TB. Kampala is Uganda’s capital with a population of approximately 2.5 million residents, an estimated TB prevalence of 870–1000/100,000 and nearly 20% of all TB cases reported to the National TB Program [31,32]. The capital district has a government-funded health system that offers free TB diagnostic evaluation and treatment services for all patients who seek care at the public health facilities. Patients are responsible for costs associated with the clinic visits such as transportation, lodging and meals. This evaluation focused on a population of urban residents of all ages because each case detection strategy is more likely to reach people with different demographic and health seeking characteristics. For example, the HCI strategy mostly finds children younger than 15 years who live at home with their parents. On the other hand, community ACF targets adults 15 years or older who are capable of reporting symptoms and producing sputum samples for evaluation. Community ACF studies done in Kampala have shown that there is a high prevalence of undetected adult TB cases [31,33].

### Study Perspective, Audience and Timeframe

This study was conducted primarily from the societal perspective as recommended by the Panel on Cost-Effectiveness in Health and Medicine [34]. The societal perspective includes all costs borne by the health providers, patients and caregivers. In a secondary analysis, we considered the health providers’ perspective; this excludes costs borne by the patients’ and caregivers. The target audience is the TB policy decision makers in the Uganda Ministry of Health, TB programs in Africa and the World Health Organization. The study timeframe spanned 1.5 years, from January 2008 to June 2009 based on the duration of the primary ACF study that was conducted in Kampala, Uganda.

### Description of Alternative Strategies

The three alternative strategies that were evaluated were 1) Passive Case Finding (PCF) alone 2) Passive *plus* Active Case Finding (PCF+ ACF) and, 3) Passive *plus* Household Contact Investigation (PCF+ HCI).

#### Passive Case Finding

Passive case-finding (PCF), the WHO standard policy recommendation for TB case detection [1] is universally practiced by the Uganda National TB control program. Persons with TB symptoms especially chronic cough (> = 2weeks) initiate an outpatient visit to the health facility for diagnostic evaluation and treatment services. Patients are screened for active TB disease using sputum smear microscopy over a 2–3 days period on average, the detailed information on the diagnosis process is provided in supporting materials (S1 Materials).

#### Passive plus Active Case Finding

We considered a hypothetical combination strategy where ACF would be added to an existing PCF program as described above in order to identify additional TB cases in the community. ACF is a well-known case finding approach that was practiced in early 1950s and is currently used in research settings [35,36]. It is a non-conventional, provider-initiated strategy that targets individuals suspected to have active TB disease within the general community or high-risk groups that have not sought care [37]. In Uganda, ACF has been performed by health care workers (HCWs) using door-to-door cough surveys [33]. The HCWs or trained volunteers in ACF perform a series of activities including: 1) travel to communities and visit participants’ homes 2) conduct short cough surveys from door-to-door to identify persons with cough > = 2 weeks 3) collect two sputum specimens for bacteriologic testing in the laboratory from those reporting chronic cough 4) return test results to the patients at their homes and refer those who have TB disease for care.

#### Passive Case Finding plus Household Contact Investigation

We also evaluated a hypothetical combination of HCI and the existing PCF strategy. Household contact investigation (HCI) is a targeted form of active case finding that aims to identify additional TB cases among household contacts of confirmed index TB cases. Although HCI is currently recommended it is rarely practiced in Uganda or other parts of Africa except in some research settings. In the ideal HCI situation, the health care workers screens all household members, defined as persons sharing meals and residing under the same roof with the index TB case [38]. The standard diagnostic protocol includes screening children and adult contacts with or without symptoms. The average household in Kampala city is estimated to have roughly four people so those would need to be evaluated in a given home [39]. A more detailed description of this strategy is provided in supplementary information (S1 Materials).

### Effectiveness Measure and Data Sources

The effectiveness measure was the number of true TB cases detected. This is an intermediate outcome that is of interest to the TB program health providers who are involved in case detection. A payoff of 1 was assigned for a true TB cases detected and a payoff of zero was assigned for a true negative, false positive and a false negative case. For the PCF+HCI strategy, a payoff of 2 was assigned for any true positive case to reflect an additional case detected through HCI efforts.

The effectiveness data were obtained from three main sources: a primary study of ACF conducted in Kampala, National TB program data for PCF and published studies for HCI [36,40,41]. Expert opinions were elicited in case data were unavailable from the main sources. The Uganda National TB Program data on the number of people screened, TB tests performed and number of active TB cases diagnosed were abstracted from clinic and laboratory registries for the period of January 2008 to June 2009. The overall data quality was good, with a few missing values which were filled in using pharmacy records and duplicate copies of patient treatment cards.

### Decision Model and Assumptions

**Fig 1:**
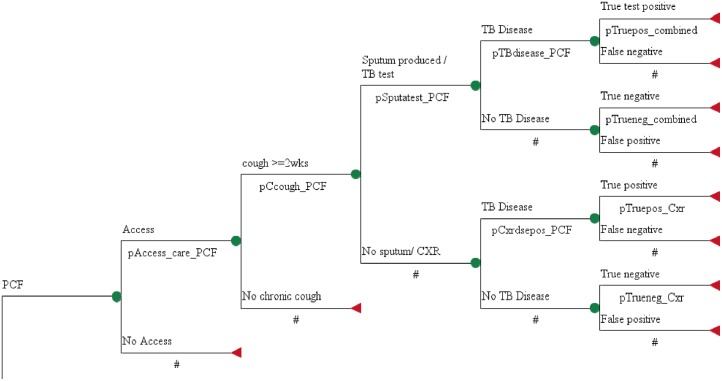
Decision Tree for the Passive Case Finding Strategy.

The decision model was structured based on the detection phase of TB disease which involves pre-diagnosis evaluation and diagnosis at the health facility. The tree begins with three choices of strategies for TB case detection: PCF alone, PCF+ACF, and PCF+ HCI. In PCF, we assumed that 57% of potential suspects access the public health system based on the estimated TB case detection rate of Uganda. The HCWs screen the patients for chronic cough and identify people with cough > = 2weeks (Fig. 1). Conditional probabilities of the different events that follow are presented including the sensitivity and specificity of the TB tests (Table 1).

**Table 1:** Probabilities Estimates Used in Decision Analytic Model.

Model parameter	Description of parameters by case detection method	Base value	Source of base value	Range for sensitivity analysis	References
	Passive Case Finding				
pAccess_care_PCF	Probability of accessing care in PCF	0.57	[58]	0.25–1.00	Assumption
pCcough_PCF	Probability of chronic cough given access	0.975	Uganda TB program records 2008–09	0.78–1.00	[36,38]
pSputatest_PCF	Probability of sputum production & TB test given chronic cough	0.899	Uganda TB program records 2008–09	0.75–0.95	[59], estimated upper value
pTBdisease_PCF	Probability of TB disease given chronic cough with sputum production	0.60	Uganda TB program records 2008–09	0.20–0.75	[48,60,61],
pCxrdsepos_PCF	Probability of TB disease detected by positive CXR given chronic cough without sputum production	0.40	Expert opinion	0.30–0.70	[14]
	Active Case Finding				
pAccess_ACF	Probability of being accessed by ACF workers	0.69	[51]	0.25–1.00	[51], estimated upper value
pCcough_ACF	Probability of chronic cough given being accessed	0.039	[51]	0.02–0.40	[33,49,62]
pSputatest_ACF	Probability sputum production & TB test given chronic cough	0.804	[51]	0.65–0.90	[33,63];
pTBdisease_ACF	Probability of TB disease given chronic cough with sputum production	0.244	[51]	0.028–0.30	[6,33]
pCxrdsepos_ACF	Probability of TB disease detected by positive chest x-ray given chronic cough without sputum production	0.196	[51]	0.10–0.30	Expert opinion
	Household Contact Investigation				
pCase_TP_HCI	Probability of TB case detected from true positive smear index case	0.19	[36]	0.06–0.24	[38,41]
pCase_FP_HCI	Probability of TB case detected from false positive smear index case	0.02	Expert opinion	0–1.0	Uncertain, full range of values examined
pCase_TPcxr_HCI	Probability of TB case detected from true positive CXR index case	0.10	[7]	0–1.0	Uncertain, full range of values examined
pCase_FPcxr_HCI	Probability of TB case detected from false positive CXR index case	0.01	Expert opinion	0–1.0	Uncertain, full range of values examined
	Sensitivity and Specificity of Tests				
pTruepos_TBtest	Sensitivity of smear test	0.609	[51]	0.30- 0.80	[60,64,65]
pTrueneg_TBtest	Specificity of smear test	0.883	[51]	0.80–0.97	[64,65]
pTruepos_combined	Combined sensitivity of Smear and culture	0.776	[64]	0.61–1.0	[51], estimated upper value
pTrueneg_combined	Combined specificity of Smear and culture	1.00	[64]	0.883–1.00	[51,64],
pTruepos_Cxr	Sensitivity of CXR	0.92	[14]	0.70–0.95	Estimated lower value, [66]
pTrueneg_Cxr	Specificity of CXR	0.63	[14]	0.52–0.99	[66,67]

**Fig 2:**
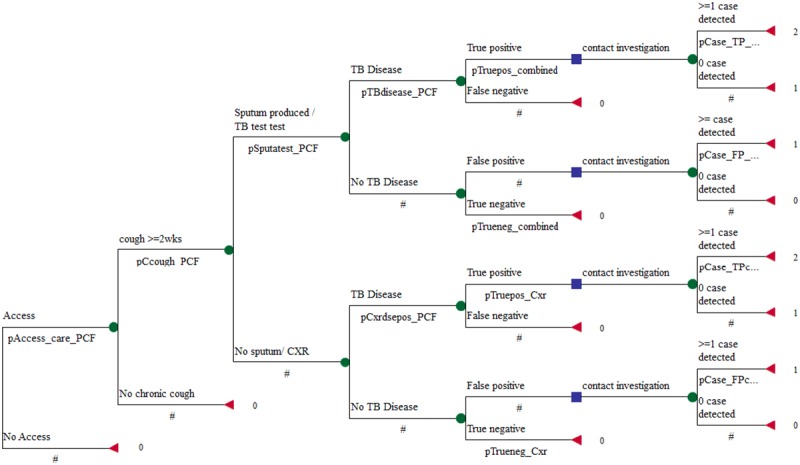
Decision Tree for Decision Tree for the Passive Case Finding *plus* Household Contact Investigation Strategy.

The PCF+HCI strategy was constructed in two parts: the detection of an index case in PCF, and the follow-up evaluation of the household contacts for TB disease. The same probabilities associated with events in the PCF alone strategy also apply to this strategy, in addition to the conditional probabilities associated with detecting a TB case among the contacts given a true or false positive index case in the household (Fig. 2). For simplicity, the HCI activities are summarized into a dichotomous outcome; detecting one or more cases and not detecting a case.

**Fig 3:**
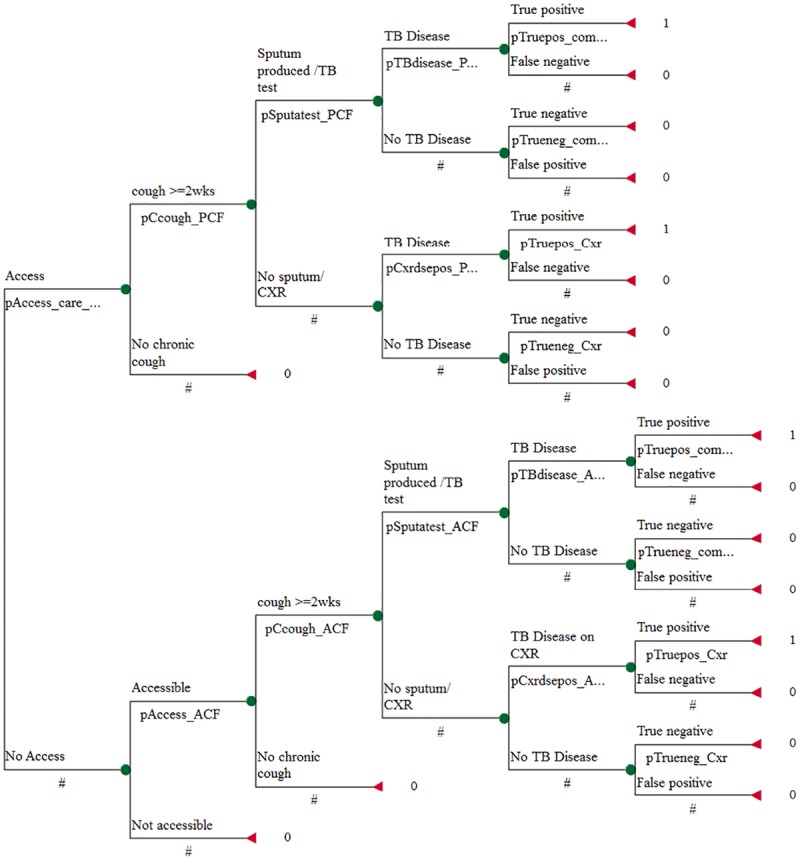
Decision Tree for the Passive Case Finding *plus* Active Case Finding Strategy.

In the PCF+ACF strategy, we considered two parallel pathways through which a TB cases can be detected. First, cases can be detected through the standard PCF path in the same way as described above. Second, additional cases can be detected by the ACF path through community door-to-door surveys as a supplement to the PCF strategy (Fig. 3). The probabilities associated with the events that follow after a person has been reached by PCF or accessed by ACF are shown in detail (Table 1).

### Uncertainty in Probability Estimates

We used the available published literature to obtain the best probability estimates for the model, but we anticipated a high degree of uncertainty in some parameters including the sensitivity and specificity of chest x-rays in persons who were unable to produce sputum, the number of TB cases that can be detected from a false smear positive case and a true or false positive chest x-ray index case since there were no published studies. The analysis was performed using TreeAge Pro 2012 software.

### Cost Estimates and Data Sources

Study costs were assessed from the societal and health provider perspectives for the period between 2008–2009 and were adjusted to 2013 U.S dollars using the consumer price index [34]. No discount rate was applied to the costs because of a short analytic horizon that was slightly over one year. Only the costs incurred during the process of diagnostic evaluation for TB disease were considered. In the combination strategies the costs from PCF were added to costs incurred in ACF or HCI respectively.

The costs were broken down into three main categories: program costs, direct medical costs and, patient and caregiver costs. Program costs were defined as costs incurred in administrative activities and personnel time [42]. Direct medical costs were defined as all costs at the point of health services such as tests, drugs and outpatient visits. Patient and caregiver costs are individual out-of-pocket expenses on meals, travel, accommodation and indirect costs due wages lost during the time of receiving health services [34,43].

Program cost data were abstracted from the national TB program budgets, research budgets and actual expense records. Medical costs were obtained from the TB program and compared with the market prices for 2008 and 2009. Patient and care giver cost data were gathered using a TB patient survey, details are provided in the supporting materials (S2 Materials and S2 Table). Overhead costs such as utilities, office space, computers, and maintenance of medical equipment were excluded from the analysis because they are ‘fixed’ and not itemized or directly allocated to a specific service in the TB program clinics [34].

Personnel cost data included time spent by nurses, clinicians and laboratory technicians involved in patient care, from screening counseling, registration through diagnosis of TB. Time was valued based on the hourly pay rate calculated from monthly salaries as paid by the Uganda Government in 2008. Administrative costs included field personnel training, transportation, phone communication, volunteer lay-workers and community mobilization in the case of ACF. Since no explicit records existed for some administrative costs for the PCF and HCI strategy, we used primary data collected from the ACF study to estimate these costs (S3 Table). For PCF, we assumed minimal costs on field transportation and no additional training costs since the existing program already has trained health workers.

Medical costs included in the analysis were costs of sputum smear and culture tests, sputum cups, gloves, cool box for specimen storage during transportation and chest x-rays in the year 2008. Costs were market-based and taken to be the same regardless of the strategy.

Patients’ and caregivers’ direct costs were estimated based on out-of pocket expenses for transportation and meals at TB clinic for evaluation for an attendance of 2.3 visits on average in PCF. Direct caregiver costs were similar to patient costs and were calculated based on 36.8% of patients who used the help of care givers during their clinic visits as reported in the cost survey (S2 Table). Patient and care giver indirect costs were estimated based on losses in wages and productivity calculated from time in travel, waiting and missed days of work during the clinic visits multiplied by Uganda’s minimum hourly wage of $0.15 (Uganda, Bureau of Statistics 2011). The minimum wage in Uganda was used as a proxy for the value of time for a person who is a non-wage earner [34] because majority of the participants were not employed, however this approach could have underestimated the costs. Patients in ACF and HCI were assumed to incur very little or no direct and indirect costs since they are mostly evaluated at home. The costs estimates for each strategy were obtained using macro-and micro-costing approaches. The program costs for ACF and HCI were much higher than the costs for PCF; however, the total patient costs were highest for PCF and low in both ACF and HCI strategies. The medical costs contributed the most to overall cost in the three strategies and were very similar across strategies similar because the same TB diagnostic tests are used (Table 2). Detailed ingredient costing for each cost category and strategy is provided in supporting information (S2 Materials, S3 Table).

**Table 2:** Summary of Cost (in 2013US$) Estimates Associated with TB Detection.

Cost category	Cost, $	Range (+/-50%)	Source of data
Program costs^a^			
PCF	7.71	3.86–11.57	Uganda TB program records 2008–09
ACF	26.88	13.44–40.32	Primary study research budgets
HCI	26.31	13.16–39.47	Primary study research budgets
PCF+ACF	34.59	17.30–51.89	
PCF+HCI	34.02	17.01–51.03	
Direct Medical ^b^			
PCF	47.14	23.57–70.71	Uganda TB program records 2008–09
ACF	47.38	23.69–71.07	Primary study research budgets
HCI	46.37	23.19–69.56	Primary study research budgets
PCF+ACF	93.52	47.26–141.78	
PCF+HCI	92.51	46.76–140.27	
Total Patient &Caregiver Costs ^c^			
PCF	28.88	14.44–43.32	TB patient cost survey
ACF	4.76	2.38–7.14	Primary study
HCI	4.76	2.38–7.14	Estimated from primary study
PCF+ACF	33.64	16.82–50.46	
PCF+HCI	33.64	16.82–50.46	
Total per Patient Costs^d^			
PCF	83.73	41.87–125.60	
ACF	79.02	39.51–118.53	
HCI	77.44	38.72–116.16	

a: Program costs include administration, transport, communication & health personnel

b: Direct medical costs include Smear tests, culture tests, CXR & consumable supplies

c: Total patient and care giver costs include direct (transportation& meals) and, Indirect costs (productivity/wages lost)

d: Estimated total per patient costs are a summation of program, direct medical and total patient-caregiver costs estimated in each strategy

### Incremental Cost-effectiveness Ratio

Incremental cost effectiveness ratios (ICERs) were calculated from dividing the difference in expected costs and difference expected number of true TB for each strategy as obtained from the decision analysis model. We compared ICERs to a threshold value of US$1102.00, two times Uganda’s annual gross domestic product (GDP) per capita as estimated by the World Bank in 2012 [44]. The threshold is defined in reference to the country’s GDP per capita following standard benchmarks proposed in international work on cost-effectiveness. When ICERs fall below the defined threshold then interventions are considered cost-effective [45,46]. This range of threshold values is generally assumed to encompass the decision makers’ willingness-to-pay for an additional unit of effectiveness in health, however much debate still surrounds the determination of an acceptable threshold. [46].

## Results

### Base Analysis

From the societal perspective, the average expected number of true TB cases per 1000 persons screened was 253 at a total average cost of $37,920, was 255 at $41,160 and was 300 at $58,500 in PCF alone, PCF+ACF and PCF+HCI respectively (See full analyzed decision tree in supporting information S1 Fig.). The incremental cost-effectiveness ratio of PCF+ACF compared to PCF alone was US$1,492.95 and of PCF+ HCI compared to PCF alone was US$443.62 per additional TB case detected (Table 3). The marginal effectiveness of HCI is an additional forty seven TB cases detected and only 2 cases for ACF. These results inform decision makers about whether to add ACF or HCI to the existing standard PCF. In reference to the set decision threshold $1,102.00, the ICER for PCF+HCI falls below the set value therefore it is a cost-effective strategy.

**Table 3:** Incremental Cost-effectiveness Ratios from the Societal Perspective Referencing PCF as a Common Baseline.

Strategy	Total cost (US$)^a^	Incremental cost	Total ^a^ effectiveness	Incremental effectiveness	Total cost/total effectiveness (ACER)	ICER ^b^ (cost per additional case detected)
PCF	37920	-	253	-	149.73	-
PCF+ACF	41160	3240	255	2	161.41	1492.95*
PCF+HCI	58500	20580	300	47	195.00	443.62*

a: Effectiveness are rounded to the nearest whole number per 1000 person screened in the target population

b: ICER- Incremental Cost-Effectiveness Ratio (incremental cost divided by incremental effectiveness)

*calculations of ICERs do not exactly match direct division of incremental cost and incremental effectiveness as shown in table because we used up to 5 significant digits for effectiveness numbers to increase precision and minimize rounding errors

### Cost-effectiveness Analysis from the Provider Perspective

In this analysis, only the costs borne by the provider were included when evaluating the expected total cost of detecting true TB cases. The expected number of true TB case per 1000 persons screened in each strategy is similar to those from the societal perspective but the costs differ. For PCF alone the total average cost of detecting 253 cases was US$21,690, in PCF+ACF for 255 cases was US$24,880 and in PCF+HCI for 300 cases is US$41,010. The incremental cost-effectiveness ratios was US$1,467.57 for PCF+ACF compared to PCF alone and was 416.35 per case detected for PCF+ HCI compared to PCF alone (Table 4). These results inform decision makers as whether to add either ACF or HCI to the existing standard PCF. In reference to the set decision threshold $1,102.00, the ICER for PCF+HCI falls below the set value therefore it is a cost-effective strategy. The results from the societal and provider perspectives were similar in direction and conclusion.

**Table 4:** Incremental Cost-effectiveness Ratios from the Provider Perspective Referencing PCF as a Common Baseline.

Strategy	Total cost (US$)^a^	Incremental cost	Total ^a^ effectiveness	Incremental effectiveness	Total cost/total effectiveness (ACER)	ICER ^b^ (cost per additional case detected)
PCF	21690	-	253	-	85.73	-
PCF+ACF	24880	3190	255	2	97.37	1467.57*
PCF+HCI	41010	19320	300	47	136.70	416.35*

a: Effectiveness are rounded to the nearest whole number per 1000 person screened in the target population

b:ICER- Incremental Cost-effectiveness Ratio (incremental cost divided by incremental effectiveness)

*calculation of ICERs do not exactly match direct division of incremental cost and incremental effectiveness as shown in table because we used up to 5 significant digits for effectiveness numbers to increase precision and minimize rounding errors

### Sensitivity Analysis

We performed one-way sensitivity analyses to explore the impact of uncertainty in the estimated probability and cost values on the base analysis. The model probabilities and costs were varied one at a time over a range of predefined plausible values (Table 5, S4 Table, and S5 Table). Probabilities were varied over extreme ranges of zero to one for TB cases detected from a true positive smear index or positive chest X-ray index in PCF+HCI due to greater uncertainty in the base values. Costs were varied over a 50% decrease and increase in the base values for the lower and upper ranges respectively as recommended by the Panel on Cost-effectiveness in Health and Medicine [34].

**Table 5:** One-way Sensitivity Analysis for Cost-effectiveness of TB Case Finding Strategies Varying Probabilities and Costs.

Strategies Compared	Incremental Cost Effectiveness Ratios (Difference in US$/Difference TB case detected)			
	PCF + ACF vs. PCF		PCF +HCI vs. PCF	
Base ICER^a^	1492.95		443.62	
Probability parameters	Low value	High value	Low value	High value
Base (Ranges: low, high)^b^				
Chronic cough in ACF	2644.88	398.61^c^	443.62	443.62
0.039 (0.02, 0.40)				
TB Disease given sputum test in ACF	5302.58 ^d^	1258.53	443.62	443.62
0.244 (0.028,0.30)				
TB test sensitivity	1808.59	1209.58	452.85	432.85
0.776 (0.61,1.0)				
CXR sensitivity	1563.39	1483.83	440.06	444.10
0.92 (0.70,0.95)				
Case detected from true positive smear index in HCI	1492.95	1492.95	1274.43^d^	87.67
0.19 (0.06,1.0)				
Costs				
Program costs in ACF	838.66^e^	2147.23	443.62	443.62
26.88 (13.44,40.32)				

^a^ICER = Incremental Cost- Effectiveness Ratio

^b^ Ranges obtained from published literature, expert opinion, or full ranges used

^**c**^ PCF+ACF becomes cost-effective at high probability of chronic cough, ICER below decision threshold of US$ 1,102.00

^d^ PCF+HCI is no longer cost effective at low probability of case detection, ICER above decision threshold

^**e**^ PCF+ACF becomes a cost-effective at low ACF program cost, ICER below decision threshold

**Fig 4:**
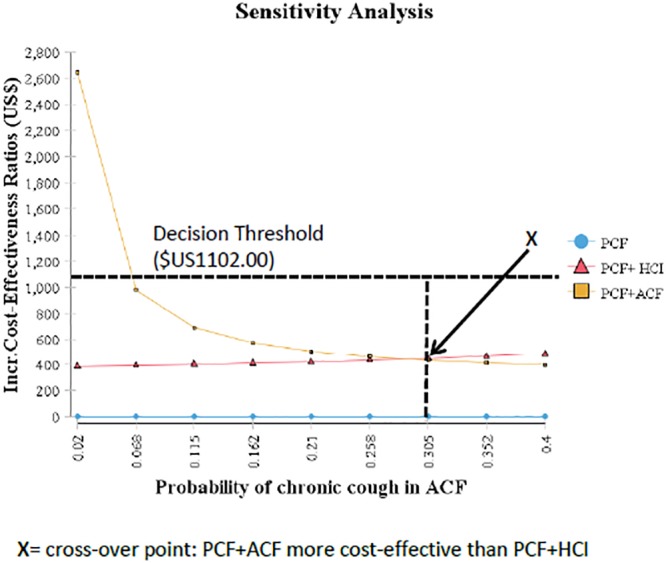
Graphic Display of One-Way Sensitivity Analysis Showing ICERs When Probability of Chronic Cough in ACF is Varied over Plausible Values from 0.04 to 0.4.

The model was most sensitive to changes in probability of one or more TB cases being detected by the HCI strategy following a true smear positive index case, the probability of chronic cough and the program cost of ACF. When the probability of one or more cases detected from HCI was varied to its lowest plausible value of 0.06, the ICER increased to $1,274.43 above the decision threshold of $1,102.00. This led to a change in the base analysis conclusion to PCF+HCI becoming no longer a cost-effective strategy (Table 5, S2 Fig.). When the probability of chronic cough in ACF was varied from 0.04 to 0.40, the ICER dropped to $398.61 per additional case detected making PCF+ACF also cost-effective at a decision threshold of $1,102.00. Moreover, a cross-over point was observed at 0.305 for the probability of chronic cough when PCF+ACF first became more cost-effective than PCF+HCI (Fig. 4). When the estimated program costs for ACF was decreased by 50% the ICER reduced to $838.66 per additional case detected making PCF+ACF cost effective strategy (Table 5, S3 Fig.). Sensitivity analyses performed on all the other parameters did not change the model conclusions. Detailed results from one-way sensitivity analyses over the range of plausible values for probabilities and costs are provided in supporting information (S4 Table and S5 Table).

## Discussion

We conducted an incremental cost-effectiveness analysis to compare PCF+ACF and PCF+HCI with PCF alone in an African city context. The results indicate that PCF+HCI is cost-effective for detecting TB cases compared to PCF alone from both the societal and health provider perspectives. The cost per additional TB case detected was $443.62 and $416.35 for the PCF+HCI strategy from the societal and provider perspective respectively. The model conclusions were sensitive to changes in the probability of detecting one or more TB cases, a 10-fold increase in the prevalence of chronic cough in ACF, and to a 50% reduction in program costs in ACF from the set baseline values. A threshold point was reached when the probability of cough reached 0.305, such that the PCF+ACF strategy was even more cost-effective than PCF+HCI. When the probability of detecting a case from a true smear positive index case in HCI was set to its lowest plausible value of 0.06, PCF+HCI was no longer cost-effective. This is not surprising because the risk of a household contact becoming a TB cases is largely driven by the infectiousness of the index case [47,48]. It is important that in the context of Uganda and much of Africa, practical challenges such as the lack of well-organized public health systems, shortage of health care personnel and limited health resources to follow up index TB cases may make the effectiveness of the PCF+HCI less achievable.

A major strength of this study is that we used probability and cost estimates mostly drawn from actual data in the Uganda National TB Program and primary studies conducted in urban Uganda. Therefore, we believe that most of the model assumptions are close enough to the real world situation. Moreover, the costs borne by patients were evaluated directly from a cost survey of patients receiving TB services from the PCF system in Kampala, Uganda; this is an addition to the existing cost literature. Furthermore, we conducted a full economic analysis comparing all the currently available TB case detection strategies from the societal perspective as recommended by the Panel on Cost-effectiveness in Health and Medicine [34].

Our study findings contrast with results from a recent study by Mupere et al. (2013) that compared the cost-effectiveness of active plus passive case finding compared to PCF alone in Kampala, Uganda and found that PCF+ACF was more cost-effective [8]. However, we cannot make direct comparison of our findings and this study because the outcome measures, the range of alternatives compared and study perspectives were different. Our study measured cost per additional true TB case detected while the study by Mupere and colleagues (2013) measured cost per additional quality-adjusted life years (QALYs), life years gained and new cases averted from the providers’ perspective [8]. Our study included PCF+HCI as a third alternative and the costs borne by the patients and care givers in addition to health providers’ costs.

Our study indicates that the PCF+ACF strategy becomes a cost-effective strategy under certain conditions. From one-way sensitivity analyses, a ten-fold increase in the prevalence of chronic cough from 4% to 40% in the population screened using ACF made the ACF +PCF strategy more cost-effective than PCF+HCI. Moreover, we observed a threshold prevalence of 30.5% where the PCF+ACF strategy first becomes the more favorable strategy. In the real world, it may be relatively rare to encounter a prevalence of chronic cough as high as 30.5% in the general population however it can occur in special situations where access to health services is very poor. For example, a study that used the ACF strategy to detect undiagnosed TB in rural Kenya found the prevalence of chronic cough to be 39% in a community where access to health care was poor [49]. Extra efforts such as periodic community mass campaigns could be undertaken by the health care workers in order to increase the likelihood of chronic cough in the screening sample [50]. Since prevalence of chronic cough emerged as an important variable in our analysis, this supports the idea that targeted screening for TB among people with chronic coughers could improve the efficiency of ACF [51].

The cost and affordability of performing ACF programs has always been a subject for ongoing debate [27]. Our study also highlighted that program costs including: personnel time, administration and transportation drove the overall cost of ACF but a. 50% reduction in the base costs dramatically reduced the cost per additional case detected by nearly US$650 in PCF+ACF thereby making the strategy cost-effective. Future research to closely evaluate ways to cut back on program costs and improve the efficiency of ACF is therefore warranted. A cost-effectiveness study done in Ethiopia that evaluated PCF alone and PCF+ACF using field extension workers instead of trained nurses found that this approach reduced the program costs by 61% and was cost-effective [29].

In Uganda, one practical approach to reducing ACF costs could be integrating TB screening services with existing community outreach health programs such as child immunization and family planning services to leverage existing human health resources. Periodic targeted screening in community gatherings such as places of worship or market places may be yet another low-cost approach to finding persons with chronic cough who need further medical evaluation. Finally, in the era of exponential growth of mobile phone technology, there is a great opportunity to reach millions of people in diverse populations with health interventions than never before. Text messaging could be an effective way to deliver mass health mobilization campaigns for periodic TB screening. In fact, there is no single ideal approach for all settings but the overarching goal should be to find as many undetected TB cases early enough at the lowest cost possible and to initiate them on effective therapy.

Of note is that mathematical modeling studies have consistently highlighted the importance of case detection and the economic benefit of ACF under specific conditions [52–55]. However, we acknowledge that we cannot make fair comparisons between results from our static decision model and those from dynamic compartmental mathematical modeling studies. Murray and Salomon (1998) used a dynamic model to estimate the maximum costs per DALY at which ACF strategies would be cost-effective when compared to the standard PCF in the different global regions over a 30 year period. For the Sub-Saharan Africa region, they found that the cost of detecting a TB case by ACF using symptomatic screening wasUS$56 per DALY gained and could reduce the number of new cases of TB by 17 million between 1998 and 2050 [52]. These findings suggested that ACF is indeed associated with remarkable future health benefits which were not demonstrated by the short-term assessment in our study [54].

Policy decision makers should view cost-effectiveness results in light of other context-specific factors, such as the burden of disease, the patient mix in the target population, ethical and equity concerns. For instance, if access to health care is deemed to be a serious problem in a given setting, then community ACF may be the only equitable way to reach the undetected cases. For example Uganda’s capital, Kampala city has five administrative divisions all of which have at least one slum setting with poor and vulnerable populations who are at high risk for TB and may lack access to health care. A study conducted in Kisenyi slum in Kampala found a high prevalence of 18% undetected TB cases among people who reported chronic cough [33]. The ideal policy decision would be to implement all the three case finding strategies in order to maximize the potential for cases detection. However in the real world, using PCF+HCI is an optimal strategy that should be prioritized and regularly re-evaluated for efficiency according to changes in the disease epidemiology and other population dynamics.

### Limitations

The findings in our study should be interpreted in light of some limitations. First, we used a static model to estimate the effectiveness of the strategies as measured by the number of true TB cases detected, this a short-term benefit. Our model did not account for potential future benefits from implementing the strategies such as the new TB cases averted by interrupting ongoing TB transmission and death prevented as a result of earlier detection. This could have led to underestimation of the overall effectiveness and cost-effectiveness of the ACF and HCI strategies in the medium to long-term period.

Second, we did not use generic effectiveness measures such Quality-Adjusted life Years (QALYs) or Disability—Adjusted Life Years (DALYs) therefore our study findings comparisons could be limited to only to studies with similar measures. However, the choice of our study effectiveness measure was guided by its relevance to answering the research question posed coupled with the immediate interest of the TB program decision makers in local context. Additionally, we used the country’s 2012 GDP as the decision threshold for willingness-to-pay under the assumption that policy makers would pay the same amount for an additional TB case detected as for an additional QALY gained or DALY averted. Currently, there is no standard benchmark, but we speculate that that the willingness-to-pay per TB case detected may be lower; hence our study finding could reflect a conservative estimate of the cost-effectiveness of the intervention strategies. A recent study that measured the value of ACF demonstrated that 2-year campaigns were highly cost-effective at less than per capita GDP when the cost per TB case detected was directly converted to cost per Disability-Life Years (DALYs) averted [56]. The most appropriate benchmark for willingness-to-pay per TB case detected is not yet clearly defined therefore more research work is needed.

Finally, our model does not explicitly account for changes in the prevalence of TB disease in the general population over time. This limits the evaluation of the potential performance of the case detection strategies at varying levels of prevalence. However, we assume that the model results hold true for the estimated prevalence of 330/100,000 in Uganda and similarly high TB prevalence areas in Africa [57].

### Conclusions

Under our baseline assumptions, HCI is more cost effective than ACF when implemented in the context of the existing PCF program for TB case detection from the societal and provider perspectives. PCF+HCI cost US$1,049 less than PCF+ACF to detect one additional true TB case. Therefore, implementation of household contact investigations as a part of the recommended TB control strategy should be prioritized.

## Supporting Information

S1 Materials Description of Alternative Strategies. (PDF)

S2 Materials Description of Cost Measurements and Data Collection Methods. (PDF)

S1 Table Composition and Credentials of Expert Opinion Team. (PDF)

S2 Table Summary of TB Patients Cost Survey Results. (PDF)

S3 Table Detailed Cost Estimation and Valuation of Resources for PCF, ACF and HCI Strategies Based on ACF Study and National TB Program Data. (PDF)

S4 Table Detailed One-way Sensitivity Analysis for Cost-effectiveness of TB Case Finding Strategies Varying Model Probabilities. (PDF)

S5 Table Detailed One-way Sensitivity Analysis for Cost-effectiveness of TB Case Finding Strategies Varying Costs. (PDF)

S1 Fig Decision Model Showing Expected Values and Optimal Case Finding Strategy. (TIF)

S2 Fig Graphical Display of Sensitivity Analysis of Probability of Detecting a TB Case in HCI. (TIF)

S3 Fig Graphical Display of Sensitivity Analysis of Program Costs for ACF. (TIF)

## References

[1] World Health Organization (2011) Global Tuberculosis Report Geneva, Switzerland.

[2] Ministry of Health (2010) Ministry of Health Manual of the National TB and Leprosy Programme.

[3] Laxminarayan R, Klein E, Dye C, Floyd K, Darley S, et al (2007) Economic Benefit of Tuberculosis Control. Washington, D.C: The World Bank.

[4] Zarocostas J (2010) A third of world’s tuberculosis cases remain undetected, says WHO. BMJ 341: c6396 10.1136/bmj.c6396 21068123

[5] Ayles H, Muyoyeta M, Du Toit E, Schaap A, Floyd S, et al. (2013) Effect of household and community interventions on the burden of tuberculosis in southern Africa: the ZAMSTAR community-randomised trial. Lancet. 10.1016/S0140-6736(13)61131-9 23915882

[6] Corbett EL, Bandason T, Duong T, Dauya E, Makamure B, et al (2010) Comparison of two active case-finding strategies for community-based diagnosis of symptomatic smear-positive tuberculosis and control of infectious tuberculosis in Harare, Zimbabwe (DETECTB): a cluster-randomised trial. Lancet 376: 1244–1253. 10.1016/S0140-6736(10)61425-0 20923715 PMC2956882

[7] Dasgupta K, Schwartzman K, Marchand R, Tennenbaum TN, Brassard P, et al (2000) Comparison of cost-effectiveness of tuberculosis screening of close contacts and foreign-born populations. Am J Respir Crit Care Med 162: 2079–2086. 11112118 10.1164/ajrccm.162.6.2001111

[8] Mupere E, Schiltz NK, Mulogo E, Katamba A, Nabbuye-Sekandi J, et al (2013) Effectiveness of active case-finding strategies in tuberculosis control in Kampala, Uganda. Int J Tuberc Lung Dis 17: 207–213. 10.5588/ijtld.12.0160 23317956

[9] Nienhaus A, Schablon A, Costa JT, Diel R (2011) Systematic review of cost and cost-effectiveness of different TB-screening strategies. BMC Health Serv Res 11: 247 10.1186/1472-6963-11-247 21961888 PMC3196701

[10] Shrestha RK, Mugisha B, Bunnell R, Mermin J, Hitimana-Lukanika C, et al (2006) Cost-effectiveness of including tuberculin skin testing in an IPT program for HIV-infected persons in Uganda. Int J Tuberc Lung Dis 10: 656–662. 16776453

[11] Kowada A, Takahashi O, Shimbo T, Ohde S, Tokuda Y, et al (2008) Cost effectiveness of interferon-gamma release assay for tuberculosis contact screening in Japan. Mol Diagn Ther 12: 235–251. 18652520 10.1007/BF03256289

[12] Pooran A, Booth H, Miller RF, Scott G, Badri M, et al (2010) Different screening strategies (single or dual) for the diagnosis of suspected latent tuberculosis: a cost effectiveness analysis. BMC Pulm Med 10: 7 10.1186/1471-2466-10-7 20170555 PMC2837635

[13] Manabe YC, Hermans SM, Lamorde M, Castelnuovo B, Mullins CD, et al (2012) Rifampicin for continuation phase tuberculosis treatment in Uganda: a cost-effectiveness analysis. PLoS One 7: e39187 10.1371/journal.pone.0039187 22723960 PMC3377630

[14] van Cleeff MR, Kivihya-Ndugga LE, Meme H, Odhiambo JA, Klatser PR (2005) The role and performance of chest X-ray for the diagnosis of tuberculosis: a cost-effectiveness analysis in Nairobi, Kenya. BMC Infect Dis 5: 111 16343340 10.1186/1471-2334-5-111 PMC1326228

[15] Kowada A, Deshpande GA, Takahashi O, Shimbo T, Fukui T (2010) Cost effectiveness of interferon-gamma release assay versus chest X-ray for tuberculosis screening of BCG-vaccinated elderly populations. Mol Diagn Ther 14: 229–236. 10.2165/11538610-000000000-00000 20799765

[16] Menzies NA, Cohen T, Lin HH, Murray M, Salomon JA (2012) Population health impact and cost-effectiveness of tuberculosis diagnosis with Xpert MTB/RIF: a dynamic simulation and economic evaluation. PLoS Med 9: e1001347 10.1371/journal.pmed.1001347 23185139 PMC3502465

[17] Andrews JR, Lawn SD, Rusu C, Wood R, Noubary F, et al (2012) The cost-effectiveness of routine tuberculosis screening with Xpert MTB/RIF prior to initiation of antiretroviral therapy: a model-based analysis. AIDS 26: 987–995. 10.1097/QAD.0b013e3283522d47 22333751 PMC3517815

[18] Vassall A, van Kampen S, Sohn H, Michael JS, John KR, et al (2011) Rapid diagnosis of tuberculosis with the Xpert MTB/RIF assay in high burden countries: a cost-effectiveness analysis. PLoS Med 8: e1001120 10.1371/journal.pmed.1001120 22087078 PMC3210757

[19] Resch SC, Salomon JA, Murray M, Weinstein MC (2006) Cost-effectiveness of treating multidrug-resistant tuberculosis. PLoS Med 3: e241 16796403 10.1371/journal.pmed.0030241 PMC1483913

[20] Floyd K, Hutubessy R, Kliiman K, Centis R, Khurieva N, et al (2012) Cost and cost-effectiveness of multidrug-resistant tuberculosis treatment in Estonia and Russia. Eur Respir J 40: 133–142. 10.1183/09031936.00169411 22362862

[21] Fitzpatrick C, Floyd K (2012) A systematic review of the cost and cost effectiveness of treatment for multidrug-resistant tuberculosis. Pharmacoeconomics 30: 63–80. 10.2165/11595340-000000000-00000 22070215

[22] Okello D, Floyd K, Adatu F, Odeke R, Gargioni G (2003) Cost and cost-effectiveness of community-based care for tuberculosis patients in rural Uganda. Int J Tuberc Lung Dis 7: S72–79. 12971657

[23] Floyd K, Skeva J, Nyirenda T, Gausi F, Salaniponi F (2003) Cost and cost-effectiveness of increased community and primary care facility involvement in tuberculosis care in Lilongwe District, Malawi. Int J Tuberc Lung Dis 7: S29–37. 12971652

[24] Moalosi G, Floyd K, Phatshwane J, Moeti T, Binkin N, et al (2003) Cost-effectiveness of home-based care versus hospital care for chronically ill tuberculosis patients, Francistown, Botswana. Int J Tuberc Lung Dis 7: S80–85. 12971658

[25] Sinanovic E, Floyd K, Dudley L, Azevedo V, Grant R, et al (2003) Cost and cost-effectiveness of community-based care for tuberculosis in Cape Town, South Africa. Int J Tuberc Lung Dis 7: S56–62. 12971655

[26] Khan MA, Walley JD, Witter SN, Imran A, Safdar N (2002) Costs and cost-effectiveness of different DOT strategies for the treatment of tuberculosis in Pakistan. Directly Observed Treatment . Health Policy Plan 17: 178–186. 12000778 10.1093/heapol/17.2.178

[27] Nishikiori N, Van Weezenbeek C (2013) Target prioritization and strategy selection for active case-finding of pulmonary tuberculosis: A tool to support country-level project planning. BMC Public Health 13: 97 10.1186/1471-2458-13-97 23374118 PMC3602078

[28] Murray CJ, Salomon JA (1998) Expanding the WHO tuberculosis control strategy: rethinking the role of active case-finding. Int J Tuberc Lung Dis 2: S9–15. 9755959

[29] Datiko DG, Lindtjorn B (2010) Cost and cost-effectiveness of smear-positive tuberculosis treatment by Health Extension Workers in Southern Ethiopia: a community randomized trial. PLoS One 5: e9158 10.1371/journal.pone.0009158 20174642 PMC2822844

[30] Government of Uganda: Minisrty of Health (2010) HEALTH SECTOR STRATEGIC PLAN III, 2010/11–2014/15

[31] Guwatudde D, Zalwango S, Kamya MR, Debanne SM, Diaz MI, et al (2003) Burden of tuberculosis in Kampala, Uganda. Bull World Health Organ 81: 799–805. 14758406 PMC2572356

[32] Uganda Ministry of Health (2010) National TB and Leprosy Program Annual Report.

[33] Sekandi JN, Neuhauser D, Smyth K, Whalen CC (2009) Active case finding of undetected tuberculosis among chronic coughers in a slum setting in Kampala, Uganda. Int J Tuberc Lung Dis 13: 508–513. 19335958 PMC2842997

[34] Gold MR, Siegel JE, Russell LB, Weinstein MC (1996) Cost-effectiveness in Health and Medicine New York: Oxford University Press.

[35] Shapiro AE, Golub JE (2012) A systematic review of active case-finding strategies in risk groups for tuberculosis and the relationship to number needed to screen Report to WHO. Center for Tuberculosis Research, Johns Hopkins.

[36] Shapiro AE, Variava E, Rakgokong MH, Moodley N, Luke B, et al (2012) Community-based targeted case finding for tuberculosis and HIV in household contacts of patients with tuberculosis in South Africa. Am J Respir Crit Care Med 185: 1110–1116. 10.1164/rccm.201111-1941OC 22427532 PMC5448579

[37] Golub JE, Mohan CI, Comstock GW, Chaisson RE (2005) Active case finding of tuberculosis: historical perspective and future prospects. Int J Tuberc Lung Dis 9: 1183–1203. 16333924 PMC4472641

[38] Guwatudde D, Nakakeeto M, Jones-Lopez EC, Maganda A, Chiunda A, et al (2003) Tuberculosis in household contacts of infectious cases in Kampala, Uganda. Am J Epidemiol 158: 887–898. 14585767 10.1093/aje/kwg227 PMC2869090

[39] UBOS (2011) Uganda Demographic and Health Survey 2011 Uganda.

[40] Lodi S, Del Amo J, d’Arminio Monforte A, Abgrall S, Sabin C, et al (2012) Risk of tuberculosis following HIV seroconversion in high-income countries. Thorax. 10.1136/thoraxjnl-2012-202544 23117980

[41] Morrison J, Pai M, Hopewell PC (2008) Tuberculosis and latent tuberculosis infection in close contacts of people with pulmonary tuberculosis in low-income and middle-income countries: a systematic review and meta-analysis. Lancet Infect Dis 8: 359–368. 10.1016/S1473-3099(08)70071-9 18450516

[42] Johns B, Baltussen R, Hutubessy R (2003) Programme costs in the economic evaluation of health interventions. Cost Eff Resour Alloc 1: 1 12773220 10.1186/1478-7547-1-1 PMC156020

[43] Drummond MF, Sculper MJ, Torrance GW, O’Brien BJ, Stoddart GL (2005) Methods for the Economic Evaluation of Health Care Programmes. New York: Oxford University Press, Inc

[44] World Bank (2013) GDP Per capitaUganda: (current US$).

[45] Hutubessy R, Chisholm D, Edejer TT (2003) Generalized cost-effectiveness analysis for national-level priority-setting in the health sector. Cost Eff Resour Alloc 1: 8 14687420 10.1186/1478-7547-1-8 PMC320499

[46] Shillcutt SD, Walker DG, Goodman CA, Mills AJ (2009) Cost effectiveness in low- and middle-income countries: a review of the debates surrounding decision rules. Pharmacoeconomics 27: 903–917. 10.2165/10899580-000000000-00000 19888791 PMC2810517

[47] Whalen CC, Zalwango S, Chiunda A, Malone L, Eisenach K, et al (2011) Secondary attack rate of tuberculosis in urban households in Kampala, Uganda. PLoS One 6: e16137 10.1371/journal.pone.0016137 21339819 PMC3038854

[48] Eang MT, Satha P, Yadav RP, Morishita F, Nishikiori N, et al (2012) Early detection of tuberculosis through community-based active case finding in Cambodia . BMC Public Health 12: 469 10.1186/1471-2458-12-469 22720878 PMC3489610

[49] van’t Hoog AH, Laserson KF, Githui WA, Meme HK, Agaya JA, et al (2011) High prevalence of pulmonary tuberculosis and inadequate case finding in rural western Kenya. Am J Respir Crit Care Med 183: 1245–1253. 10.1164/rccm.201008-1269OC 21239690

[50] Shargie EB, Morkve O, Lindtjorn B (2006) Tuberculosis case-finding through a village outreach programme in a rural setting in southern Ethiopia: community randomized trial. Bull World Health Organ 84: 112–119. 16501728 10.2471/blt.05.024489 PMC2626531

[51] Sekandi JN, List J, Luzze H, Yin XP, Dobbin K, et al (2014) Yield of undetected tuberculosis and human immunodeficiency virus coinfection from active case finding in urban Uganda. Int J Tuberc Lung Dis 18: 13–19. 10.5588/ijtld.13.0129 24365547 PMC5454493

[52] Murray CJ, Salomon JA (1998) Modeling the impact of global tuberculosis control strategies. Proc Natl Acad Sci U S A 95: 13881–13886. 9811895 10.1073/pnas.95.23.13881 PMC24946

[53] Borgdorff MW, Floyd K, Broekmans JF (2002) Interventions to reduce tuberculosis mortality and transmission in low- and middle-income countries. Bull World Health Organ 80: 217–227. 11984608 PMC2567749

[54] Currie CS, Floyd K, Williams BG, Dye C (2005) Cost, affordability and cost-effectiveness of strategies to control tuberculosis in countries with high HIV prevalence. BMC Public Health 5: 130 16343345 10.1186/1471-2458-5-130 PMC1361804

[55] Dowdy DW, Chaisson RE (2009) The persistence of tuberculosis in the age of DOTS: reassessing the effect of case detection. Bull World Health Organ 87: 296–304. 19551238 10.2471/BLT.08.054510 PMC2672581

[56] Azman AS, Golub JE, Dowdy DW (2014) How much is tuberculosis screening worth? Estimating the value of active case finding for tuberculosis in South Africa, China, and India. BMC Med 12: 216 25358459 10.1186/s12916-014-0216-0 PMC4224697

[57] World Health Organization (2013) Global Tuberculosis Report. Geneva, Switzerland.

[58] World Health Organization (2012) Global Tuberculosis Report Geneva, Switzerland.

[59] Kiwuwa MS, Charles K, Harriet MK (2005) Patient and health service delay in pulmonary tuberculosis patients attending a referral hospital: a cross-sectional study. BMC Public Health 5: 122 16307685 10.1186/1471-2458-5-122 PMC1310609

[60] Dasgupta K, Menzies D (2005) Cost-effectiveness of tuberculosis control strategies among immigrants and refugees. Eur Respir J 25: 1107–1116. 15929967 10.1183/09031936.05.00074004

[61] Nakiyingi L, Kateete DP, Ocama P, Worodria W, Sempa JB, et al (2012) Evaluation of in-house PCR for diagnosis of smear-negative pulmonary tuberculosis in Kampala, Uganda. BMC Res Notes 5: 487 10.1186/1756-0500-5-487 22947399 PMC3497582

[62] Ayles H, Schaap A, Nota A, Sismanidis C, Tembwe R, et al (2009) Prevalence of tuberculosis, HIV and respiratory symptoms in two Zambian communities: implications for tuberculosis control in the era of HIV. PLoS One 4: e5602 10.1371/journal.pone.0005602 19440346 PMC2680044

[63] Tadesse T, Demissie M, Berhane Y, Kebede Y, Abebe M (2011) Two-thirds of smear-positive tuberculosis cases in the community were undiagnosed in Northwest Ethiopia: population based cross-sectional study. PLoS One 6: e28258 10.1371/journal.pone.0028258 22164256 PMC3229563

[64] Levy H, Feldman C, Sacho H, van der Meulen H, Kallenbach J, et al (1989) A reevaluation of sputum microscopy and culture in the diagnosis of pulmonary tuberculosis. Chest 95: 1193–1197. 2656111 10.1378/chest.95.6.1193

[65] Aber VR, Allen BW, Mitchison DA, Ayuma P, Edwards EA, et al (1980) Quality control in tuberculosis bacteriology. 1. Laboratory studies on isolated positive cultures and the efficiency of direct smear examination. Tubercle 61: 123–133. 6777919 10.1016/0041-3879(80)90001-x

[66] Tattevin P, Casalino E, Fleury L, Egmann G, Ruel M, et al (1999) The validity of medical history, classic symptoms, and chest radiographs in predicting pulmonary tuberculosis: derivation of a pulmonary tuberculosis prediction model. Chest 115: 1248–1253. 10334135 10.1378/chest.115.5.1248

[67] Cohen R, Muzaffar S, Capellan J, Azar H, Chinikamwala M (1996) The validity of classic symptoms and chest radiographic configuration in predicting pulmonary tuberculosis. Chest 109: 420–423. 8620716 10.1378/chest.109.2.420

