# Supplementary material for: Cost-Effectiveness Analysis of Community Active Case Finding and Household Contact Investigation for Tuberculosis Case Detection in Urban Africa
Source: PLoS One. 2015 Feb 6;10(2):e0117009. doi: 10.1371/journal.pone.0117009 (PMC4319733; doi:10.1371/journal.pone.0117009)
Supplement: S1 Materials — (PDF) [file pone.0117009.s001.pdf]

Materials S1: Description of Alternative Strategies

Three alternative strategies for TB case detection were evaluated; the choice for PCF and HCI was based on the WHO recommended standard policy for case detection [1]. Although HCI is current recommended it not widely practiced in Africa. Community ACF is a well-known case finding approach that was practiced in early 1950s and is currently used in research settings [2, 3]. Since PCF is already an established standard, this study evaluated add-on strategies in order to improve effectiveness. The strategies to be compared are listed below.

1) Passive Case Finding (PCF)

2) Passive *plus* Active Case Finding (PCF+ ACF)

3) Passive *plus* Household Contact Investigation (PCF+ HCI)

Passive Case Finding

Passive case-finding (PCF) is the standard facility-based approach for detecting pulmonary TB cases and is practiced by the Uganda national TB control program. Persons with TB symptoms initiate the visit to the health facility for diagnostic evaluation and treatment services delivered through outpatient care by healthcare workers (HCWs). Patients commonly present with chronic cough; defined as self-reported cough lasting 2 or more weeks at the time of first contact with the health system.

When patients first arrive at the clinic they are screened for symptoms by a nurse; those with chronic cough are then referred to a clinician for physical examination, followed by collection of one spot sputum sample for acid- fast bacilli (AFB) smear microscopy examination and culture tests. The tests are performed free of charge in public health facilities. On the second or subsequent days, the patients return to the clinic with the second 'early morning' sputum sample for drop-off to the laboratory for testing. Depending on the patient load at the clinic, patients may wait at the clinic to receive final test results or may be required to return, on average it takes 2-3 days to complete the diagnostic process [4]. According to the standard diagnostic protocol, TB disease is confirmed if one or both tests are positive on smear and/or culture test. If a patient is unable to produce sputum, then a diagnosis is made based on chest X-rays and clinical findings. Chest X-rays results are usually obtained on the same day of the procedure.

The AFB test is simple, cheap but the culture test may require more expertise and is more expensive; both tests are available in public health system in Uganda. The sensitivity of the AFB smear test ranges between 45-63% and specificity of 79- 98% in the patient population [5]. In this study we used results from parallel testing with smear and culture, therefore the combined sensitivity of 77.6% for the two tests is considered ([6]. The sensitivity of a chest X-rays ranges from 69- 92% while the specificity ranges from 52-99% [7]. The costs associated with PCF mainly arise from the transportation, meals, caregiver costs, waiting and travel time during the multiple visits during diagnostic evaluation.

Passive plus Active Case Finding

In this strategy, ACF would be added to an existing PCF program described above in order to identify additional TB cases. ACF is a non-conventional, provider-initiated strategy to identify symptomatic individuals within the general community or high-risk groups who are suspected to have active TB disease but have not sought care [8]. In Uganda, ACF has been performed in research settings using door-to-door cough screening to identify TB suspects [9].

The health care workers (HCWs) or trained volunteers in ACF make at least 3 visits and perform a series of activities including 1) travel to the communities and visit participants in their homes 2) conduct brief cough interviews lasting 5 minutes on average, to identify persons with chronic cough 3) collect two sputum specimens for AFB smear and culture testing in the laboratory in two visits 4) return test results to the patients and refer for care if found to have TB disease. The same standard diagnostic protocol is followed as described in the PCF strategy. The provider costs associated with ACF strategy arise from the personnel time spent in travel, the community outreach activities and transportation during the home visits. Patients' costs are very minimal in ACF; those who are unable to produce sputum have to travel to the clinic to receive chest x-rays.

Passive Case Finding plus Household Contact Investigation

Household contact investigation (HCI) is a targeted form of active case finding strategy that aims to identify additional TB cases among household contacts of a confirmed index active TB cases. The strategy evaluated is a combination of HCI and the existing PCF standard strategy from which index TB cases are generated. Therefore the success of this strategy is driven by how well index cases are followed up by the health system after diagnosis. HCI is not standard practice but it is performed in some research settings in Kampala, Uganda. In the ideal HCI situation, the health care workers screen all household members defined as, persons sharing meals and residing under same roof with the index TB case [10]. The standard diagnostic protocol includes screening those with and without symptoms. For purposes of this economic evaluation we assume that those without symptoms and children who cannot produce sputum will be evaluated using chest x-rays. Although we recognize that more complex diagnostic algorithm exist including gastric aspiration for diagnosis of TB in children, we took the most pragmatic approach for this analysis.

On average a household in Kampala has four persons that would be evaluated in a given home [11]. We assumed that all true and false TB index cases from PCF would lead to household contact investigation. Some cases in the households could be missed by HCI but it is safe to assume that they will eventually be detected by the PCF strategy. Assuming that contacts are evaluated in their homes, the costs incurred in HCI would be very similar to those of ACF except for personnel time of travel from house to house. The travel time should be slightly less in HCI because more people would be evaluated in one place at a time.

Reference List

1. World Health Organization, *Global Tuberculosis Report* 2011: Geneva, Switzerland.
2. Shapiro, A.E., Golub, J. E., *A systematic review of active case-finding strategies in risk groups for tuberculosis and the relationship to number needed to screen. Report to WHO*, 2012, Center for Tuberculosis Research, Johns Hopkins.
3. Shapiro, A.E., et al., *Community-based targeted case finding for tuberculosis and HIV in household contacts of patients with tuberculosis in South Africa.* Am J Respir Crit Care Med, 2012. **185**(10): p. 1110-6.
4. Mauch, V., et al., *Free tuberculosis diagnosis and treatment are not enough: patient cost evidence from three continents.* Int J Tuberc Lung Dis, 2013. **17**(3): p. 381-7.
5. Albert, H., *Economic analysis of the diagnosis of smear-negative pulmonary tuberculosis in South Africa: incorporation of a new rapid test, FASTPlaqueTB, into the diagnostic algorithm.* Int J Tuberc Lung Dis, 2004. **8**(2): p. 240-7.
6. Levy, H., et al., *A reevaluation of sputum microscopy and culture in the diagnosis of pulmonary tuberculosis.* Chest, 1989. **95**(6): p. 1193-7.
7. Dasgupta, K. and D. Menzies, *Cost-effectiveness of tuberculosis control strategies among immigrants and refugees.* Eur Respir J, 2005. **25**(6): p. 1107-16.
8. Golub, J.E., et al., *Active case finding of tuberculosis: historical perspective and future prospects.* Int J Tuberc Lung Dis, 2005. **9**(11): p. 1183-203.
9. Sekandi, J.N., et al., *Active case finding of undetected tuberculosis among chronic coughers in a slum setting in Kampala, Uganda.* Int J Tuberc Lung Dis, 2009. **13**(4): p. 508-13.
10. Guwatudde, D., et al., *Tuberculosis in household contacts of infectious cases in Kampala, Uganda.* Am J Epidemiol, 2003. **158**(9): p. 887-98.
11. UBOS, *Uganda Demographic and Health Survey 2011 Uganda*, 2011.
