# Supplementary material for: Cost-Effectiveness Analysis of Community Active Case Finding and Household Contact Investigation for Tuberculosis Case Detection in Urban Africa
Source: PLoS One. 2015 Feb 6;10(2):e0117009. doi: 10.1371/journal.pone.0117009 (PMC4319733; doi:10.1371/journal.pone.0117009)
Supplement: S2 Materials — (PDF) [file pone.0117009.s002.pdf]

**Materials S2: Description of Cost Measurements and Data Collection Methods S2**

## Cost Measurements

Study costs are assessed from the societal and health provider perspectives for the period of 2008-2009 and were adjusted to 2013 U.S. dollars using the consumer price index and the general inflation rates in the reference year as recommended by the panel on cost-effectiveness [1]. No discount rate was applied because the analytic horizon was very short (~one year). Only costs incurred during the process of diagnostic evaluation for detection of a TB case were considered. In the combined strategies the costs from PCF are added to those incurred in ACF or HCI.

The costs are broken down into three main categories: program costs, medical costs and, patient and caregiver costs. Program costs refer to costs incurred at the administrative levels outside the point of delivery and personnel costs [2]. Medical costs refer to all costs at the point where health care is delivered such as tests, drugs and outpatient visits. Patient and caregiver costs are individual out-of-pocket expenses on meals, travel, accommodation and indirect costs due wages lost during the time of receiving services [1, 3].

### *Program Cost Data Collection*

For this analysis, we considered all costs related to delivery of all three case finding interventions during a period of 18 months. Most cost information was abstracted from the national TB program budgets and actual cost records, research budgets and expense records. Efforts were made to adhere to guidelines as stipulated in the literature [4]. Program costs included in this analysis are personnel time and administrative activities (training, community mobilization, lay workers, transportation, and communication). Overhead costs such as utilities, custodial services, buildings, office space, computers, and maintenance of medical equipment were excluded when valuing resources. The justification for exclusion is that overhead costs are considered ‘fixed’ and not itemized or directly allocated to a specific service in the TB program clinics [1].

*Personnel costs:* We considered the personnel time spent by nurses, clinicians and laboratory technicians involved in patient care, from screening counseling, registration through diagnosis of TB. Time was valued based on the hourly pay rate calculated from monthly salaries as paid by the government of Uganda in 2008. The hourly rates are US $1.13 for nurses; $0.76 for laboratory technicians and $2.75 for a clinician. A full week of work is equivalent to 40 hours in the formal employment sector in Uganda. The total personnel costs were obtained from multiplying the hourly rate by estimated patient contact time with each personnel and summing up the costs. Based on our cost survey data, patients identified by PCF require 2.3 outpatient visits to complete the diagnosis process. For ACF and HCI, we estimated that on

average, one hour is spent to complete each patient evaluation in the field based on time motion surveys done in a random sample of study participants in the primary ACF study.

*Transportation costs:* Transportation costs were incurred when HCWs travelled to the communities to perform case finding activities in ACF or HCI. Minimal costs on transportation arose from program-related activities in PCF. The cost of transportation were obtained from the annual program budget for PCF and research budget for ACF and scaled to 18 months then divided by the total number of people screened in each program to obtain a per person cost. The cost of HCI was estimated as two thirds of the ACF program since HCI would involve slightly less travel given that more people would be evaluated within a household.

*Training costs:* Training costs arose from time spent on extra training of HCWs to enable them to perform the various activities in ACF and HCI. For example, during the primary ACF study, ten HCWs were trained for a total period of one month spread over the 18-month study period. We assume that similar training would be necessary for HCI. No additional training would be needed for PCF since the existing program personnel have the necessary skills to perform the routine case finding activities at the clinics. The total training costs were obtained from research project expense records and were divided by the number of persons screened to obtain the per person training cost.

*Community mobilization and lay health volunteer costs:* In order to perform ACF successfully, we conducted health education campaigns involving community members and their local leaders prior to the door-to-door surveys. Costs were incurred in transportation refunds for the leaders, provision of refreshments at meetings and hiring public address systems. Two lay volunteers were identified for each village to travel with the study team each day to ensure that all eligible homes were visited. There were 18 villages therefore 36 volunteers were involved in the study; each volunteer received a reimbursement fee for his/her time at a flat rate of US$2.00/ day. Costs were obtained from the project budget and verified by multiplying the daily pay rate, number of volunteers and number of days that each worked. This cost was only incurred in ACF.

*Communication costs:* During the ACF and HCI projects, phone communication among study teams, the laboratory, study participants, and the community volunteers were vital for the smooth running of the field activities. The total communication cost for ACF is obtained from the records as expenditure on phone services as an average of US$120.00 per month over the 18 months period. Due to lack of specific records for PCF and HCI, the cost was estimated as fraction of ACF costs: 50% for HCI and 30% for PCF. The justification is that these programs would have a lower scale of activities requiring phone communication.

***Medical Costs***

Medical costs included in the analysis are costs of smear test, culture test, sputum cups, gloves, and chest x-rays as of 2008. Costs are market-based and are the same regardless of the case finding strategy. The costs were $3.00 for two smear tests, $15.00 for culture test and $8.00 for chest x-rays per person during the study period in 2008 and 2009. These costs were adjusted to 2013 US dollars.

***Patient and Caregiver Costs***

*Patient and caregiver direct costs*: We estimated total direct patient and caregiver costs including out-of pocket costs from transportation and meals while attending TB clinic visits for diagnostic evaluation using a patient survey. Patients detected through the PCF strategy incurred an average total cost of $17.26 because of the need to travel at least two times to the clinic before a diagnosis is confirmed. Direct caregiver costs would be similar to patient costs except that they are calculated based on the proportion of patients who reported to have used care givers. On average ACF and HCI patients would spend $4.29; this applies only to those who would need to travel to the clinic for a chest x-ray.

*Patient and care giver indirect costs*: Patient indirect costs are estimated based on patient time spent in travel, waiting time, the diagnostic evaluation process at the clinic visit and lost days of work. In ACF and HCI, very minimal or no indirect costs are incurred since people are evaluated in their homes. Based on survey results, patients and caregivers in PCF lost a total of 73.5 hours on average during the diagnostic evaluation process. The time lost was multiplied by Uganda's minimum hourly wage of $0.15 per hour (Uganda, Bureau of Statistics 2011) to obtain the indirect cost. We valued patients' and caregivers' time using the minimum wage in Uganda as a proxy for the value of time for a person who is a non-wage earner [1].

***Description of Data Collection in Primary Studies***

Primary data were collected during a community active case finding survey and a patient cost survey conducted in Kampala, Uganda. A cross-sectional survey was conducted in 2008-2009 to detect active TB cases in urban communities in persons aged 15 years and older. Trained research nurses and social workers visited homes, administered questionnaires, identified persons with chronic cough, collected sputum specimens for laboratory examinations, returned results and referred patients for appropriate care. On average, three visits were made to the patient homes by the study teams.

Over the 18-month study period 5,102 participants were enrolled from nearly 4,400 households. The prevalence of chronic cough was 3.9%. Among chronic coughers who were tested for TB, 39 (24.4%) had TB disease detected through ACF. Patients were referred to the public health clinics for treatment. Detailed results of the study are published elsewhere [Sekandi, et al, IJTLD, 2013 in press]. The results of

this primary study were used to generate path probabilities and effectiveness data for the ACF strategy in the model.

### *Description of TB Patient Cost Survey*

This patient cost survey is an addition to the literature on costs associated with the case detection phase for a PCF patient as there is no published primary study for urban Ugandan TB patients. Adult patients 15 years and older, with a confirmed TB diagnosis and already receiving treatment were selected to participate in the cost survey. The survey was conducted in two TB clinics in Kampala; a referral hospital clinic where most of the national TB program data were obtained and another public health center IV (designated clinic for comprehensive TB patient management). The selection was based on the logic that a high patient load is served at these urban clinics. Patients who had at least completed two weeks of TB treatment were recruited from November to December 2012.

A structured questionnaire was used to collect detailed information regarding all costs incurred by the TB patients and their care givers for each diagnosis related visit during the period when the patient was being evaluated for TB up to the time of initiating treatment (see questionnaire in Appendix D). Information about direct and indirect costs was gathered. Direct costs were defined as out-of-pocket expenditures, including transportation fees, food and drinks for the patients and their caregivers. Indirect included travel time, waiting time and absence from work by the patient or caregivers. Caregivers time was estimated from time spent when a family member/friend escorted the patient to the outpatient clinic.

### *Published Literature*

We utilize published medical literature to obtain parameter values that were not available from the primary data and the national TB program database. Most of the parameter estimates for HCI studies were obtained from published studies performed in Uganda [5, 6] and elsewhere in Africa [7]. Ranges of values used in sensitivity analyses were also obtained from published literature. Estimates from higher order studies such as randomized trials, meta-analyses and prospective observational studies were preferred and, priority given to those conducted in Uganda or in African settings when available.

### *Expert Opinion*

In situations when data were insufficient from the primary sources and/or good quality published literature studies were not available, expert opinions were elicited. This approach to estimating probability values and other study parameters is well established [1]. A team of 5 clinical experts were used to estimate the probability of a positive chest x-ray when a person has chronic cough but fails to produce sputum in PCF, the probability of detecting a case from a false positive smear index, true positive

chest X-ray index and false positive chest X-ray index. The level of uncertainty around these values is therefore likely to be very high. More details of the process of generating estimates by the expert panel and the experts' credentials are provided. The credentials of the expert panel members are provided in the Table S1.

**Reference List**


1. Gold, M.R., Siegel, J.E, Russell, L.B., Weinstein, M.C., Cost-effectiveness in Health and Medicine. 1996, New York: Oxford University Press.
2. Johns, B., R. Baltussen, and R. Hutubessy, Programme costs in the economic evaluation of health interventions. Cost Eff Resour Alloc, 2003. 1(1): p. 1.
3. Drummond, M.F., Sculper, M. J., Torrance, G.W., O'Brien, B.J. ,Stoddart, G.L., Methods for the Economic Evaluation of Health Care Programmes. 2005, New York: Oxford University Press, Inc.
4. Haddix, C.A., Teutsch, M. S., Corso, P. S, Prevention Effectiveness: A Guide to Decision Analysis and Economic Evaluation. Second ed. 2003, New York, NY: Oxford University Press, Inc.
5. Guwatudde, D., et al., Tuberculosis in household contacts of infectious cases in Kampala, Uganda. Am J Epidemiol, 2003. 158(9): p. 887-98.
6. Whalen, C.C., et al., Secondary attack rate of tuberculosis in urban households in Kampala, Uganda. PLoS One, 2011. 6(2): p. e16137.
7. Shapiro, A.E., et al., Community-based targeted case finding for tuberculosis and HIV in household contacts of patients with tuberculosis in South Africa. Am J Respir Crit Care Med, 2012. 185(10): p. 1110-6.
