# Supplementary material for: Cost-Effectiveness Analysis of Community Active Case Finding and Household Contact Investigation for Tuberculosis Case Detection in Urban Africa
Source: PLoS One. 2015 Feb 6;10(2):e0117009. doi: 10.1371/journal.pone.0117009 (PMC4319733; doi:10.1371/journal.pone.0117009)
Supplement: S1 Table — (PDF) [file pone.0117009.s003.pdf]

Table S1 Composition and Credentials of Expert Opinion Team

Expert	Qualifications	Area of Expertise	Work Settings
Dr. Christopher Whalen	M.D, MS	TB & HIV Epidemiology research	U.S & Uganda
Dr. Juliet Sekandi	MBChB, MS	TB &HIV health services research	Uganda
Dr. Achilles Katamba	MBChB, MS, PhD	TB program and surveillance	Uganda
Dr. William Worodria	MBChB, MMED, PhD	TB & HIV clinical research	Uganda
Dr. Sarah Zalwango	MBChB, MPH	TB/HIV clinical & community research	Uganda
Dr. Jonathan Golub	PhD	TB and HIV epidemiologic research	U.S, Brazil, South Africa
