# Supplementary material for: Cost-Effectiveness Analysis of Community Active Case Finding and Household Contact Investigation for Tuberculosis Case Detection in Urban Africa
Source: PLoS One. 2015 Feb 6;10(2):e0117009. doi: 10.1371/journal.pone.0117009 (PMC4319733; doi:10.1371/journal.pone.0117009)
Supplement: S2 Table — (PDF) [file pone.0117009.s004.pdf]

Table S2 Summary of TB Patients Cost Survey Results

Patient Characteristics N=103	Frequency (percent)
**Sex**	
Male	60 (58)
Female	43 (42)
**Mean Age (SD)**	32 (10.04)
**Division of Residence**	
Rubaga	36 (35)
Nakawa	9 (9)
Central	8 (8)
Kawempe	26 (25)
Makindye	9 (9)
Other	15(14)
**Clinic of diagnosis**	
National TB clinic, Mulago	75 (73)
Other	28 (27)
**Chest X-Ray**	
Yes	94 (91)
No	9 (9)
**Employed**	
Yes	59(57)
No	44(43)
**Patient costs**	
Median monthly income (IQR) in US$[a]	2.8 (0-120)
Mean monthly income (SD) in US$[b]	76 (132)
Mean number of clinic visits (SD)	2.5 (1.16)
Mean travel cost (SD) in US$[c] One way	1.21 (0.94)
Mean travel time in minutes (SD)	58 (44)
Mean waiting time (SD) in hours	5.4 (4.7)
Mean expense on meals	0.99 (1.12)
Mean hours of work (SD)	6.2 (6)
Mean days off work (SD)	1.1 (1.5)
Transportation type	
Public transport	54 (52)
Private	10 (10)
Boda boda	34 (33)
Other	5 (5)
Caregivers	
**Company of care givers**	
Yes	28 (27)
No	75(72)
**Median number of caregivers**	1 (0-1)
**Help with childcare while away at clinic visit**	
Yes	9 (9)
No	94(91)
**Hired help while away at clinic visit**	
Yes	16 (17)
No	81 (83)
**Mean total cost of help/childcare (SD)**	4.55 (11.49)
