# Supplementary material for: Cost-Effectiveness Analysis of Community Active Case Finding and Household Contact Investigation for Tuberculosis Case Detection in Urban Africa
Source: PLoS One. 2015 Feb 6;10(2):e0117009. doi: 10.1371/journal.pone.0117009 (PMC4319733; doi:10.1371/journal.pone.0117009)
Supplement: S3 Table — (PDF) [file pone.0117009.s005.pdf]

Table 3S Detailed Cost Estimation and Valuation of Resource Consumption in PCF, ACF and HCI Strategies Based on ACF and National TB Program Data

Program Costs	PCF	ACF	HCI	PCF+ACF	PCF+HCI
Personnel	2.55	11.33	7.94	13.88	10.49
Admin supervision	0.6	0.71	0.01	1.31	0.61
Field transport	0.45	1.76	0.62	2.21	1.07
Training	0	0.1	0.03	0.1	0.03
Community sensitization	0	0.35	0.06	0.35	0.06
Phone communication	0.032	0.423	0.1	0.455	0.14
Printing, copying, office supplies	0.748	0.882	0.311	1.63	1.06
Community volunteers	0	0.35	0	0.35	0.00
**Total**	4.38	14.74	9.071	20.285	13.46
**Total adjusted for inflation to 2013US$**	7.71	28.02	15.95	35.73	23.66
**Direct Medical Costs**					
2 smear tests	3.00	3.00	3.00	6.00	6.00
2 culture test	15.00	15.00	15.00	30.00	30.00
Chest X-ray	8.00	8.00	8.00	16.00	16.00
Consumable supplies	0.75	0.88	0.31	1.63	0.48
**Total**	26.75	26.88	26.31	53.63	52.48
**Total adjusted for inflation to 2013US$**	47.17	47.38	46.37	94.55	92.50
**Direct Patient Costs**					
Avg Transportation for 2.3 visits @ $1.58, 2-way	3.63				
Avg Transportation for 1 visits @ $1.58, 2-way		1.58	1.58	5.21	5.21
Meals for 2.3 visits @ $0.99	2.28				
Meals for 1 visit @ $0.99		0.99	0.99	3.27	3.27
Avg care giver costs	5.91	0	0		5.91
Child care/hired help/day	4.55	1.5	1.5	6.05	6.05
**Total**	16.37	4.07	4.07		20.44
**Indirect Patient Costs /Productivity Losses**					
Avg total patient time lost in outpatient care (36.75)					
Avg total patient &C/giver time lost (73.5hrs)	11.03	0.45	0.45	11.48	11.48
Avg total patient &C/giver time lost in ACF/HHCI (3 hrs)					
Min. wage hourly rate in Uganda ($0.15)					
**Total**	27.4	4.52	4.52	31.92	28.87
**Patient total cost adjusted for inflation to 2013US$**	28.88	4.76	4.76	33.64	33.64
