# Supplementary material for: Cost-Effectiveness Analysis of Community Active Case Finding and Household Contact Investigation for Tuberculosis Case Detection in Urban Africa
Source: PLoS One. 2015 Feb 6;10(2):e0117009. doi: 10.1371/journal.pone.0117009 (PMC4319733; doi:10.1371/journal.pone.0117009)
Supplement: S4 Table — (PDF) [file pone.0117009.s006.pdf]

**Table S4 Detailed One-way Sensitivity Analysis for Cost-effectiveness of TB Case Finding Strategies Varying Model Probabilities**

| Strategies Compared | Incremental Cost Effectiveness Ratios (US$/ TB case detected) | | | |
|---|---|---|---|---|
| | PCF + ACF vs. PCF | | PCF +HCI vs. PCF | |
| Base ICER[a] | 1492.95 | | 443.62 | |
| Probability parameters<br>Base (Ranges: low, high)[b] | For low value | For high value | For low value | For high value |
| Access to persons by HCW in ACF<br>0.69 (0.25,1.0) | 1492.95 | 1492.95 | 443.62 | 443.62 |
| Access to health service in PCF<br>0.57 (0.25, 1.0) | 1492.95 | 0.00 | 443.62 | 443.62 |
| Chronic cough in ACF<br>0.039 (0.02, 0.40) | 2644.88 | **398.61**[c] | 443.62 | 443.62 |
| Chronic cough in PCF<br>0.975 (0.78,1.0) | 1492.95 | 1492.95 | 443.62 | 443.62 |
| Produce sputum in ACF<br>0.804 (0.65, 0.86) | 1488.51 | 1495.07 | 443.62 | 443.62 |
| Produce sputum in PCF<br>0.899 (0.75,0.95) | 1492.95 | 1492.95 | 505.43 | 424.90 |
| TB Disease given sputum test in ACF<br>0.244 (0.028,0.30) | **5302.58** [d] | 1258.53 | 443.62 | 443.62 |
| TB Disease given sputum test in PCF<br>0.60 (0.20,0.75) | 1492.95 | 1492.95 | 506.37 | 436.69 |
| Combined TB test sensitivity<br>0.776 (0.61,1.0) | 1808.59 | 1209.58 | 452.85 | 432.85 |
| CXR positive in ACF<br>0.40 (0.10,0.30) | 1644.74 | 1357.25 | 443.62 | 443.62 |
| CXR positive in PCF<br>0.40 (0.30, 0.70) | 1492.95 | 1492.95 | 443.20 | 444.82 |
| CXR sensitivity<br>0.92 (0.70,0.95) | 1563.39 | 1483.83 | 440.06 | 444.10 |
| Case detected from true positive smear index in HCI<br>0.19 (0.06,1.0) | 1492.95 | 1492.95 | **1274.43**[e] | 87.67 |

[a] ICER= Incremental Cost- Effectiveness Ratio
[b] Ranges obtained from published literature, expert opinion, or full ranges used
[c] PCF+ACF becomes a cost-effective strategy at ICER $398.61
[d] Large change, > 3 times increase from base ICER
[e] PCF+HCI is no longer cost effective at ICER 1274.43
