# Supplementary material for: Cost-Effectiveness Analysis of Community Active Case Finding and Household Contact Investigation for Tuberculosis Case Detection in Urban Africa
Source: PLoS One. 2015 Feb 6;10(2):e0117009. doi: 10.1371/journal.pone.0117009 (PMC4319733; doi:10.1371/journal.pone.0117009)
Supplement: S5 Table — (PDF) [file pone.0117009.s007.pdf]

Table S5 Detailed One-way Sensitivity Analysis for Cost-effectiveness of TB Case Finding Strategies Varying Costs

Strategies Compared	Incremental Cost Effectiveness Ratios (US$/ TB case detected)			
	PCF + ACF vs. PCF		PCF +HCI vs. PCF	
Base ICER[a]	1492.95		433.62	
Cost parameters<br>Base (Ranges low, high)[b]	For low value	For high value	For low value	For high value
Program costs in PCF<br>7.71 (3.86,11.57)	1492.95	1492.95	443.62	443.62
Program costs in ACF<br>26.88 (13.44,40.32)	**838.66**[c]	**2147.23**	443.62	443.62
Program costs in HCI<br>26.31 (13.16,39.47)	1492.95	1492.95	368.29	519.01
Medical cost in PCF<br>46.14 (23.57,70.71)	1492.95	1492.95	443.62	443.62
Medical cost in ACF<br>47.38 (23.69, 71.07)	**1366.66**[d]	**1619.24**	443.62	443.62
Medical cost in HCI<br>46.37 (23.19,69.56)	1492.95	1492.95	310.83	576.47
Total patient costs in PCF<br>28.88(14.44,43.32)	1492.95	1492.95	443.62	443.62
Total patient costs in ACF<br>4.76 (2.38,7.14)	1480.26	1505.64	443.62	443.62
Total patient costs in HCI<br>4.76 (2.38,7.14)	1492.95	1492.95	429.99	457.25
Cost of TB test<br>31.72 (15.86,47.58)	1523.17	1462.73	443.62	443.62
Cost of CXR<br>14.10 (7.05,21.15)	1509.53	1476.39	443.62	443.62

[a] ICER= Incremental cost- effectiveness ratio
[b] Range estimated as +/-50% of base values in the model
[c] PCF+ACF becomes cost-effective at ICER 838.66
[d] More than $200 change in ICER when medical costs in ACF vary
