# Supplementary figures and images for: Cost-Effectiveness Analysis of Community Active Case Finding and Household Contact Investigation for Tuberculosis Case Detection in Urban Africa
Source: PLoS One. 2015 Feb 6;10(2):e0117009. doi: 10.1371/journal.pone.0117009 (PMC4319733; doi:10.1371/journal.pone.0117009)

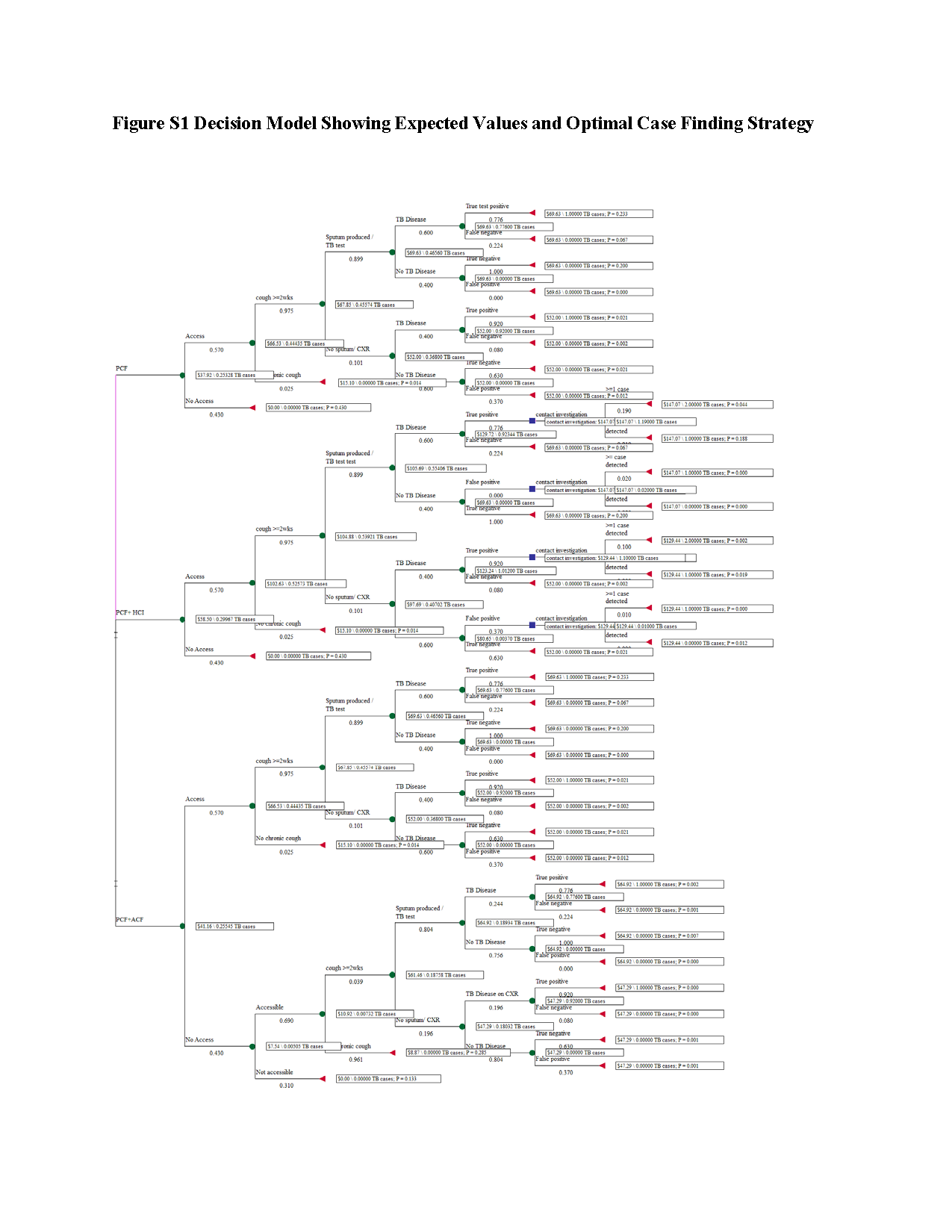

Supplement: S1 Fig — (TIF) [file pone.0117009.s008.tif]

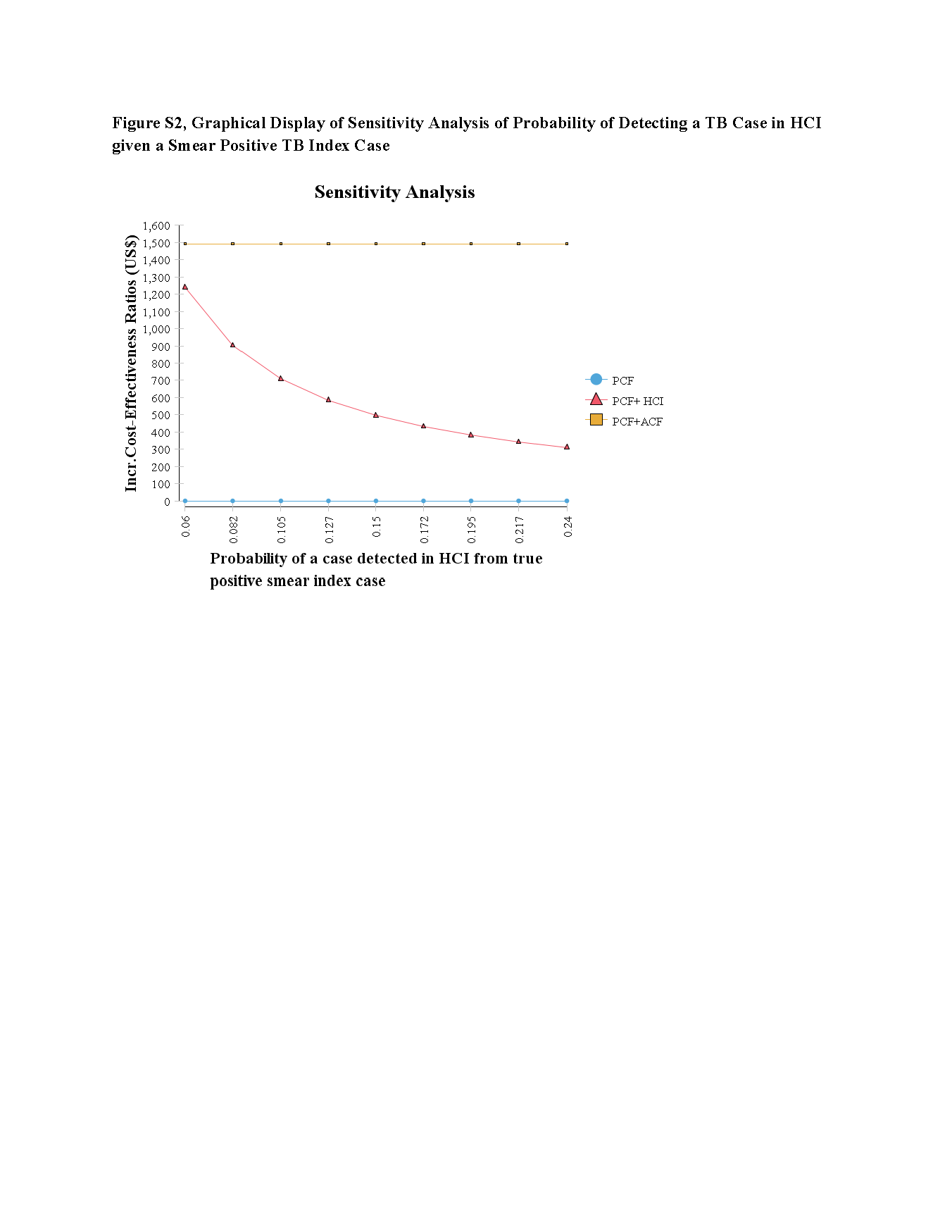

Supplement: S2 Fig — (TIF) [file pone.0117009.s009.tif]

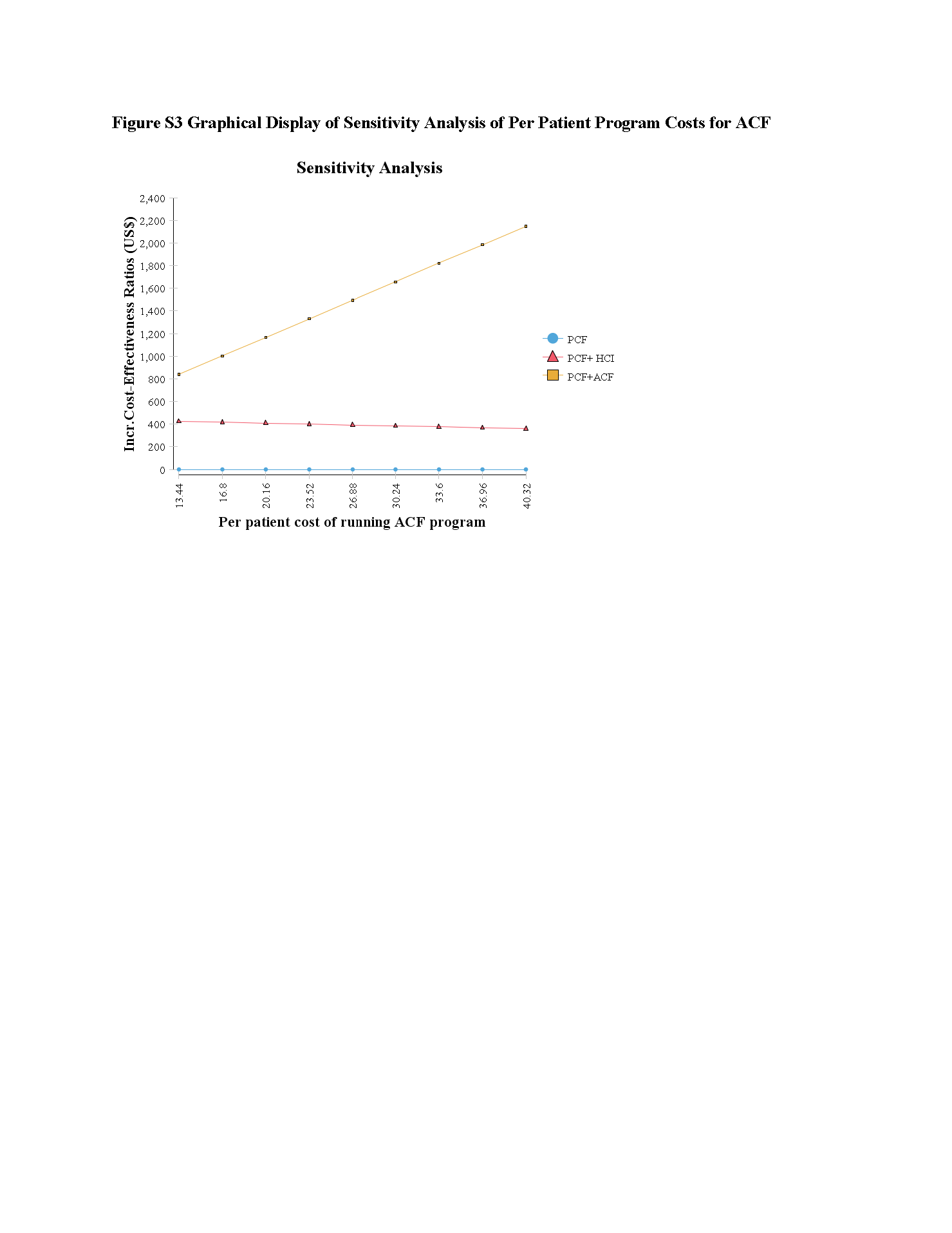

Supplement: S3 Fig — (TIF) [file pone.0117009.s010.tif]
